# Ventilator-associated pneumonia in patients assisted by veno-arterial extracorporeal membrane oxygenation support: Epidemiology and risk factors of treatment failure

**DOI:** 10.1371/journal.pone.0194976

**Published:** 2018-04-13

**Authors:** Adrien Bouglé, Camille Bombled, Dimitri Margetis, Guillaume Lebreton, Charles Vidal, Marine Coroir, David Hajage, Julien Amour

**Affiliations:** 1 Sorbonne Université, Assistance Publique - Hôpitaux de Paris (AP-HP), Department of Anesthesiology and Critical Care Medicine, Institute of Cardiology, Pitié-Salpêtrière Hospital, Paris, France; 2 Sorbonne Université, UMR INSERM 1166, IHU ICAN, Pitié-Salpêtrière Hospital, Paris, France; 3 Sorbonne Université, Assistance Publique - Hôpitaux de Paris (AP-HP), Department of Thoracic and Cardiovascular Surgery, Institute of Cardiology, Pitié-Salpêtrière Hospital, Paris, France; 4 Sorbonne Université, Assistance Publique - Hôpitaux de Paris (AP-HP), Department of Biostatistics, Public Health and Medical Information, Pitié-Salpêtrière Hospital, Paris, France; 5 Sorbonne Paris Cité, UMR 1123 ECEVE, Université Paris Diderot, Paris, France; 6 INSERM, UMR 1123 ECEVE, Paris, France; University of Colorado Denver, UNITED STATES

## Abstract

**Introduction:**

Ventilator-associated pneumonia (VAP) is frequent in Intensive Care Unit (ICU) patients. In the specific case of patients treated with Veno-Arterial Extracorporeal Membrane Oxygenation Support (VA-ECMO), VAP treatment failures (VAP-TF) have been incompletely investigated.

**Methods:**

To investigate the risk factors of treatment failure (VAP-TF) in a large cohort of ICU patients treated with VA-ECMO, we conducted a retrospective study in a Surgical ICU about patients assisted with VA-ECMO between January 1, 2013, and December 31, 2014. Diagnosis of VAP was confirmed by a positive quantitative culture of a respiratory sample. VAP-TF was defined as composite of death attributable to pneumonia and relapse within 28 days of the first episode.

**Results:**

In total, 152 patients underwent ECMO support for > 48h. During the VA-ECMO support, 85 (55.9%) patients developed a VAP, for a rate of 60.6 per 1000 ECMO days. The main pathogens identified were *Pseudomonas aeruginosa* and Enterobacteriaceae. VAP-TF occurred in 37.2% of patients and was associated with an increased 28-day mortality (Hazard Ratio 3.05 [1.66; 5.63], P<0.001), and VA-ECMO assistance duration (HR 1.47 [1.05–2.05], P = 0.025).

Risk factors for VAP-TF were renal replacement therapy (HR 13.05 [1.73; 98.56], P = 0.013) and documentation of *Pseudomonas aeruginosa* (HR 2.36 [1.04; 5.35], P = 0.04).

**Conclusions:**

VAP in patients treated with VA-ECMO is associated with an increased morbidity and mortality. RRT and infection by *Pseudomonas aeruginosa* appear as strong risks factors of treatment failure. Further studies seem necessary to precise the best antibiotic management in these patients.

## Introduction

As illustrated with the most recent data [1], the incidence of extracorporeal membrane oxygenation (ECMO) support has increased for over a decade. In-hospital mortality of patients assisted with veno-arterial (VA-ECMO) or veno-venous (VV-ECMO) ECMO remains high, secondary to the severity of illness leading to ECMO support, but also to frequent and severe complications, most being infectious [2]. Hence, nosocomial infections under ECMO support (from 14.8 to 75.5 infectious episodes per 1000 ECMO days) are associated with longer duration of ECMO support, mechanical ventilation, and ICU and hospital lengths of stay [3–6].

Although pneumonia appears to be the most frequent infection under ECMO, no specific data exist on this specific issue. Epidemiology, risk factors, microbiology, specific outcome or risks of treatment failure (TF) of ventilator-associated pneumonia under ECMO support remain unknown. However, severity of patients assisted with ECMO, immunosuppression secondary to extracorporeal circulation [7–9] as well as changes in antibiotics pharmacokinetics under ECMO [10–12] make us think that VAP under ECMO is a distinct entity.

To address this issue, we aimed to investigate the epidemiology, risk factors, microbiology and outcomes of VAP, and to identify risk factors of TF in a large and well-defined cohort of ICU patients treated with VA-ECMO.

## Methods

### Design and setting

This observational, retrospective and monocentric study was conducted in the Surgical Intensive Care Unit of Cardiology Institute in La Pitié-Salpêtrière University Hospital (Paris, France). We included all patients assisted with VA-ECMO and admitted in our unit.

### Standard care procedure for patients on ECMO

Procedures for implantation, daily management and weaning were realized according to the recommendations of the Extracorporeal Life Support Organization (ELSO) and are described in S1 File. For patients with peripheral cannulation, prophylactic antibiotic therapy was performed with second-generation cephalosporin as a single intravenous injection (cefazoline 2g) at the time of ECMO implantation. Patients with central cannulation received prophylactic antibiotic for the surgery (cefazoline 2 g within the hour prior to incision, then 1g every 4h), but no antibiotic prophylaxis was continued during the course of ECMO.

### Prevention of VAP

VAP prevention is based on a combination of measures: orotracheal intubation, endotracheal tube cuff pressure maintained between 20 and 30 cm H_2_O, oral chlorhexidine application four times per day, semi recumbent body positioning (30 to 45°) when possible. Sedation is patient-targeted, relying on assessments eight times per day of Richmond Agitation-Sedation Scale (RASS). Patients are started on enteral tube feeds within 48 h of intubation whenever possible.

### Definitions

VAP diagnosis included a clinical suspicion (≥ two criteria including fever > 38.5 ° C, leukocytosis > 10^9^/L or leukopenia < 4.10^8^/L, purulent tracheobronchial secretions and a new or persistent infiltrate on chest radiography), and confirmation by a positive quantitative culture of a respiratory sample: bronchoalveolar lavage fluid (significant threshold ≥10^4^ colony-forming units (CFU).mL^-1^) or plugged telescopic catheter (significant threshold ≥10^3^ CFU.mL^-1^) or quantitative endotracheal aspirate distal pulmonary secretion samples (significant threshold ≥10^6^ CFU.mL^-1^).

Ventilator-associated pneumonia under ECMO support was defined *post hoc* by 2 independent infectious experts (AB, CB) as a VAP occurring in patients assisted with VA-ECMO for more than 24 hours or withdrawn for less than 48 hours. Immunosuppression was defined as HIV, immunosuppressive therapy, or corticosteroids> 0.5 mg/kg per day for more than a month. In case of persistence, worsening or recurrence of clinical criteria, a new respiratory sample was performed. After 6 days of antibiotic therapy against the identified pathogen, and up to 48 hours after the end of antibiotic therapy, we considered a positive quantitative culture of the same pathogen as a persistence of VAP (S1 Fig). If this new respiratory sample was performed after ≥ 48 hours without antibiotic treatment, we considered positive quantitative culture of the same pathogen as a relapse of VAP. During antibiotic therapy of initial VAP, and up to 48 hours after the end of antibiotic therapy, we considered a positive quantitative culture of the new pathogen as a superinfection. After ≥ 48 hours without antibiotic treatment, we considered positive quantitative culture of the new pathogen as a new VAP. Nevertheless, because the occurrence of a new VAP, documented with a new pathogen is part of the prolonged ventilation of a severe ICU patient, and does not appear to be directly related to the presence of ECMO, we did not consider it as a treatment failure. Attributable death to VAP was defined as death under ECMO during antibiotic therapy for VAP without any other evident cause of death, or death under ECMO secondary to septic shock of pulmonary origin.

Our primary endpoint was treatment failure, a composite of attributable death to pneumonia or persistence or relapse of pneumonia within 28 days of the first episode.

### Data collection

The following information were recorded retrospectively for each patient: demographic data; clinical variables; comorbidities; reason for ECMO; duration of ECMO support, mechanical ventilation, ICU stay, and hospital stay; presence and type of infectious complications, hemorrhagic complications, digestive complications, neurologic complications and acute kidney injury.

### Ethics approval and consent to participate

The ethics committee Ile de France 5 approved the collection of data compiled in our ECMO registry (Reference B-7-15). Due to the retrospective design of the study, and in accordance with the decision of the ethics committee, no consent was needed.

### Statistical analysis

Results are expressed as number of patients (%), or mean (± Standard deviation). When indicated, 95% CIs were calculated. Influence of the occurrence of VAP on overall survival, ECMO assistance duration and mechanical ventilation duration were assessed with univariate Cox regression models, with ECMO-associated pneumonia as a time-dependant covariable. Kaplan-Meier representation of the overall survival according to ECMO-associate pneumonia occurrence was obtained using the Simon and Makuch method [13].

Univariate and multivariate Poisson regression survival models with the log of ECMO duration as an offset term were used to evaluate risk factors of ECMO-associated pneumonia. Univariate and multivariate Cox analyses were used to identify risk factors of treatment failure among patients with VAP. In both cases, all variables associated (p < 0.10) with the outcome in the univariate analysis were introduced into the multivariate model. Relative risks (RR) for Poisson model and hazard ratios (HR) for Cox model were calculated with their 95% confidence intervals (CI).

Significance was defined as p-values less than 0.05. Statistical analyses were performed using R 3.3.3 (http://www.R-project.org).

## Results

### Population

Between January 2013 and December 2014, 163 patients were assisted by VA-ECMO and retrospectively included in our database. Of these 163 patients, 11 died within the 48 first hours and were not included in the study. Demographic and clinical characteristics of the 152 patients with ECMO support > 48 hours are described in Table 1. VA-ECMO was implanted mainly for left ventricular failure (N = 66, 43.4%), for a period of 10.7 (8.6) days, with duration of mechanical ventilation of 14.4 (12.7) days and an ICU stay of 26.3 (24.8) days. Five patients were assisted with central cannulation (3,3%).

**Table 1:** Baseline characteristics.

All patients (N = 152)	Results N (%) or mean (± SD)
Age (years)	59 ± 15
Male sex	112 (73.7%)
BMI (Kg.m^-2^)	25.1 ± 6.1
SAPS 2	54.4 ± 22.3
SOFA	11 ± 5
Euroscore2	21.4 ± 15.8
Creatinine clearance (mL.min^-1^)	77 ± 36
LVEF (%)	30 ± 16.0
Hypertension	87 (57.2%)
Tobacco	72 (47.7%)
COPD	32 (21.0%)
Diabetes	35 (23.0%)
Liver cirrhosis	11 (7.2%)
Immunosuppression	42 (27.6%)
Reason for ECMO	
Left Ventricular Failure	66 (43.4%)
Right Ventricular Failure	43 (28.3%)
Biventricular Failure	34 (22.4%)
Cardiac Arrest	9 (5.9%)
ECMO implantation	
Pre cardiotomy	46 (30.3%)
Per cardiotomy	71 (46.7%)
Post cardiotomy	37 (24.2%)
Localization of implantation	
Operating Room	67 (44.4%)
ICU	84 (55.6%)
Prophylactic antibiotic at implantation	103 (69.1%)
Duration of ECMO support (days)	10.7 ± 8.6
Duration of Mechanical Ventilation (days)	14.4 ± 12.7
Duration of ICU stay (days)	26.3 ± 24.8
Duration of hospital stay (days)	42.7 ± 43.8
Infectious complications	
Bacteremia	53 (34.9%)
Ventilator-Associated Pneumonia	85 (55.9%)
Local infection of the cannulation site	37 (24.3%)
Catheter-Related Infection	11 (7.2%)
Urinary Tract Infection	5 (7.9%)
Mediastinitis	17 (11.2%)
Hemorrhagic complications	
Local hemorrhage of the cannulation site	20 (13.2%)
Surgical Site hemorrhage	41 (27.0%)
Digestive complications	
Gastrointestinal hemorrhage	23 (15.1%)
Mesenteric ischemia	8 (5.3%)
Neurologic complications	
Ischemic stroke	21 (13.9%)
Hemorrhagic stroke	9 (5.9%)
Limb ischemia	18 (11.8%)
Renal Replacement Therapy	93 (61.2%)
28-Day mortality	48 (31.6%)
ICU Mortality	69 (45.4%)

BMI: Body Mass Index; ASA: American Society of Anesthesiologists; SAPS2: Simplified Acute Physiology Score 2; LVEF: Left Ventricular Ejection Fraction; COPD: chronic obstructive pulmonary disease. Data are No. (%) of patients or mean value (± standard deviation)

### Description of ventilator-associated pneumonia under ECMO support

VAP occurred in 85 (55.9%) patients, for a rate of 60.6 per 1000 ECMO days. Diagnosis of VAP was realized with a delay of 5 [3;12] days after ICU admission, 3 [2;8] days after ECMO implantation and 3 [2–10] days after endotracheal intubation. Patients’ characteristics and diagnosis criteria are summarized in S1 and S2 Tables. *Pseudomonas aeruginosa* (n = 22, 18.2%) was the most frequent isolated pathogen (see Table 2).

**Table 2:** Pathogens isolated from respiratory samplings.

Pathogen	No (%)
**Bacteria**	
Gram-negative Pathogens	
*Enterobacteriaceae*	
*Escherichia coli*	10 (8.3)
*Enterobacter cloacae*	11 (9.0)
*Klebsiella pneumoniae*	8 (6.6)
*Klebsiella oxytoca*	3 (2.5)
*Hafnia alvei*	2 (1.6)
*Citrobacter koseri*	3 (2.5)
*Citrobacter freundii*	1 (0.8)
*Serratia marcescens*	5 (4.1)
*Proteus mirabilis*	2 (1.6)
*Neisseria sicca*	2 (1.6)
*Proteus vulgaris*	1 (0.8)
*Other Gram-negative pathogens*	
*Pseudomonas aeruginosa*	22 (18.2)
*Stenotrophomonas maltophilia*	3 (2.5)
Gram-positive Pathogens	
*Staphylococcus aureus*	7 (5.8)
*Oral streptococci*	1 (0.8)
*Streptococcus dysgalactiae*	2 (1.6)
*Enterococcus faecalis*	1 (0.8)
Other	
*Haemophilus influenzae*	7 (5.9)
Commensal oropharyngeal flora	23 (19.0)
*Bacteroides fragilis*	1 (0.8)
**Other pathogens**	
*Candida albicans*	3 (2.5)
*Candida glabrata*	1 (0.8)
*Herpes simplex virus*	2 (1.6)

**Fig 1:**
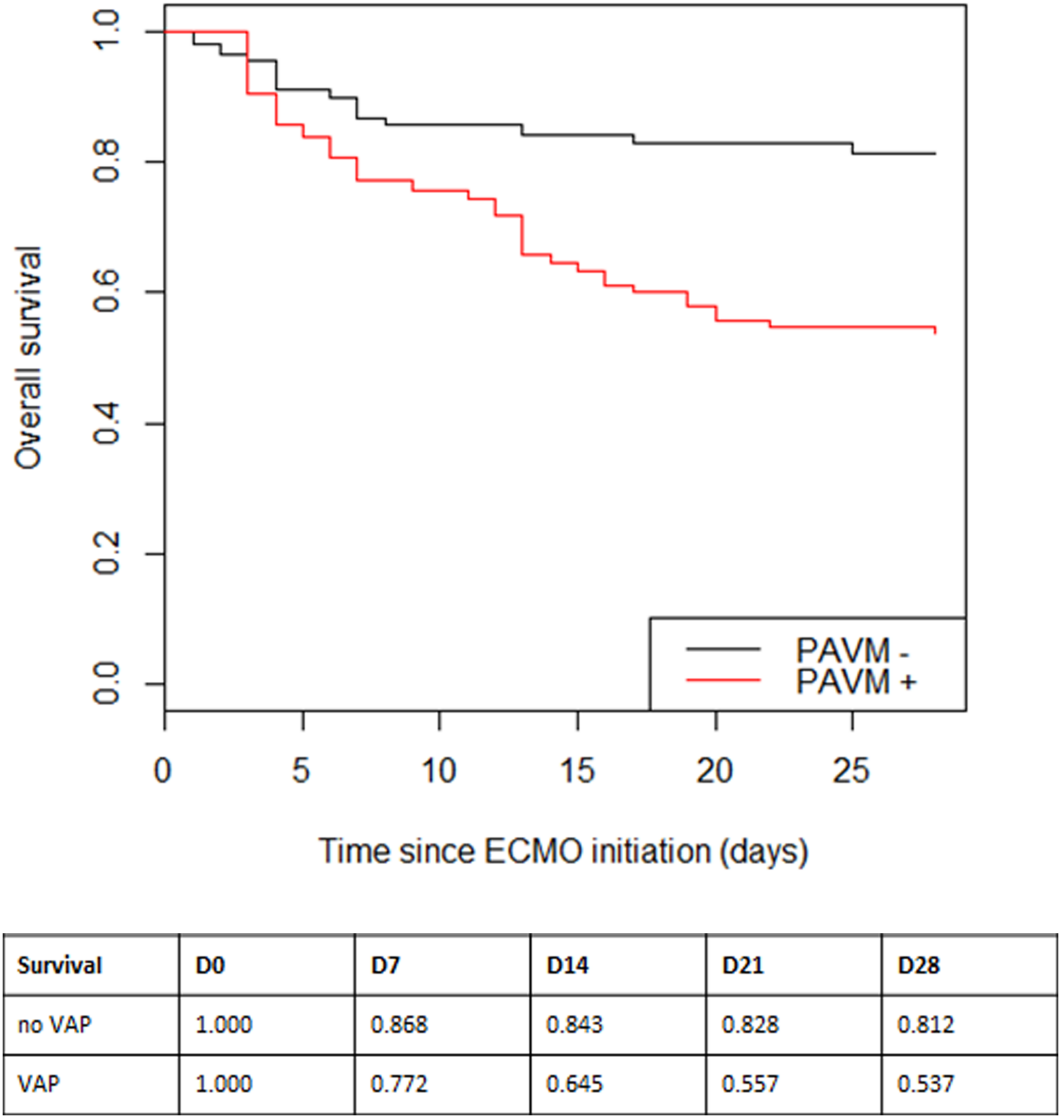
Kaplan-Meier analysis of the probability of survival according to the presence of a VAP during ECMO support. The 28-day survival was significantly lower in the VAP group (red line) in comparison to the no VAP group (black line) (nominal p < 0.001).

Occurrence of a VAP was associated with increased length of ECMO assistance duration (HR 1.47 [1.05–2.05], p = 0.025) and an increased mortality (HR 3.05 [1.66;5.63], p < 0.001) as illustrated on “Fig 1”, but not with the duration of mechanical ventilation (HR 0.86 [0.60–1.23], p = 0.406).

### Risk factors of VAP under ECMO support

In univariate analysis, risk factors for VAP were: age > 65 years old (RR [CI95%] 1.73 [1.13;2.63], p = 0.01), SOFA score at admission (1.09 [1.02;1.17], p = 0.01), history of hypertension (2.25 [1.43;3.53], p = 0.0004), active smoking (1.55 [0.96;2.49], p = 0.07) and COPD (2.15 [1.30;3.55], p = 0.003) whereas the female gender (0.44 [0.24;0.81], p = 0.008) was protective (see Table 3). Due to too many missing data, SOFA score at admission was not included in the multivariate analysis, which found only the female gender as associated with a decrease in the incidence of VAP (0.52 [0.30;0.91], p = 0.02). Due to the small number of patients with central cannulation (N = 5) present in this subgroup and therefore the insufficient power, we did perform any statistical analysis.

**Table 3:** Risk factors for VAP under ECMO.

Variable	No VAP N = 67	VAP N = 85	Univariate analysis		Multivariate analysis	
			RR [CI95%]	p	RR [CI95%]	p
Age > 65 years old	24 (35.8)	30 (35.3)	1.73 [1.13;2.63]	0.01	1.22 [0.76;1.96]	0.41
Sex (female)	24 (35.8)	16 (18.8)	0.44 [0.24;0.81]	0.008	0.52 [0.30;0.91]	0.02
Euroscore2	22.52 (16.5)	20.46 (15.2)	0.99 [0.97;1.01]	0.23		
SAPS2	48.07 (19.2)	60.34 (23.7)	1.01 [1.00;1.03]	0.08		
SOFA	9.28 (4.2)	11.75 (5.6)	1.09 [1.02;1.17]	0.01		
Diabetes mellitus	13 (19.4)	22 (25.9)	1.64 [0.91;2.97]	0.1		
Hypertension	37 (55.2)	50 (58.8)	2.25 [1.43;3.53]	0.0004	1.57 [0.93;2.67]	0.09
Active smoker	28 (42.42)	44 (51.8)	1.55 [0.96;2.49]	0.07	1.02 [0.59;1.76]	0.94
COPD	13 (19.4)	19 (22.3)	2.15 [1.30;3.55]	0.003	1.18 [0.62;2.25]	0.61
Creatinine clearance	76.3 (35.0)	76.7 (37.4)	1.00 [0.99;1.00]	0.27		
Immunosuppression	15 (22.4)	27 (31.8)	1.11 [0.66;1.86]	0.69		

SAPS2: Simplified Acute Physiology Score; SOFA: Sequential Organ Failure Assessment; COPD: chronic obstructive pulmonary disease; Data are No. (%) of patients or mean value ± standard deviation.

### Treatment failure

VAP recurrence was diagnosed in 32 patients (37.2%), with 10 persistences (11.8%), 8 relapses (9.4%), and 19 superinfections (22.3%). Gram-negative bacteria were identified in 86% of VAP recurrences, including 51% *Pseudomonas aeruginosa* and 35% Enterobacteriaceae. No clinical or biological parameters predicted the occurrence of recurrence (S2 Fig). Treatment failure was identified in 31.8% of patients (N = 27 patients). Among these patients, 92.3% (N = 24) were initially treated with appropriate antibiotics, versus 86.4% (N = 46) of the patients without treatment failure (P = 0.2765). In multivariate analysis, only renal replacement therapy at pneumonia diagnosis (HR 13.05 [1.73; 98.56], p = 0.013) and identification of *Pseudomonas aeruginosa* as the pathogen responsible for the first episode of pneumonia (HR 2.36 [1.04; 5.35], p = 0.04) were associated with treatment failure (see Table 4).

**Table 4:** Risk factors for VAP treatment failure in patients assisted with ECMO.

Variable	No VAP N = 67	VAP N = 85	Univariate analysis		Multivariate analysis	
			HR [CI95%]	p	HR [CI95%]	p
Age (years old)	57.38 (14.92)	64.26 (11.4)	1.03 [1;1.06]	0.0510	1.00 [0.97;1.04]	0.802
Sex (female)	13 (22.41%)	3 (11.11%)	0.48 [0.14;1.59]	0.2273		
BMI (Kg.m^-2^)	24.8 (4.41)	24.9 (5.8)	1.01 [0.93;1.1]	0.7432		
SOFA score	11.25 (5.47)	13.25 (5.99)	1.1 [0.97;1.25]	0.1324		
Active smoker	28 (48.28)	16 (59.26)	1.46 [0.67;3.14])	0.3386		
COPD	12 (20.69)	7 (25.93)	1.34 [0.57;3.18]	0.5008		
Liver cirrhosis	7 (12.07)	3 (11.11)	0.9 [0.27;2.99]	0.86		
Left ventricle unload	29 (50.88)	13 (48.15)	0.83 [0.39;1.77]	0.6322		
Duration of MV before VAP diagnosis	6.24 (6.64)	6.85 (7.19)	1.01 [0.96;1.06]	0.7334		
Creatinine Clearance	80.67 (37.75)	68.12 (35.98)	0.99 [0.98;1]	0.1287	1.00 [0.98;1.01]	0.431
RRT at VAP diagnosis	32 (55.17)	26 (96.3)	18.31 [2.47;135.55]	0.0044	13.05 [1.73;98.56]	0.013
Immunosuppression	19 (32.76)	8 (29.63)	0.89 [0.39;2.02]	0.7726		
Bacteremia	7 (12.07)	4 (14.81)	1.37 [0.47;3.96]	0.5639		
MDR pathogen VAP	6 (10.34)	5 (18.52)	1.53 [0.58;4.03]	0.3948		
*P*. *aeruginosa* VAP	8 (13.79)	12 (44.44)	3.02 [1.41;6.49]	0.0046	2.36 [1.04;5.35]	0.04

COPD: chronic obstructive pulmonary disease; MV: mechanical ventilation; VAP: ventilator-associated pneumonia; RRT: Renal Replacement Therapy; MDR: multidrug resistant

Data are No. (%) of patients or mean value (± standard deviation).

## Discussion

In this large retrospective cohort study in VA-ECMO patients, VAP occurs in 56% of patients, early after VA-ECMO implantation, and increases significantly the 28-day mortality. Treatment failure was identified as a frequent event, associated with the documentation of *Pseudomonas aeruginosa* and the presence of renal replacement therapy at diagnosis. The previously published studies about infectious complications under ECMO support are either pediatric patients-focused [14], from large international registries [4], or interested in all types of infections, and did not address this specific issue, whereas VAP remains the main infection in intensive care [15] as in ECMO patients [4–6].

Incidence of infections under ECMO and particularly VAP varies greatly depending the studies, ranging from 14.8 to 75.5 infectious episodes per 1000 days of ECMO [3–6]. With a rate of 60.6 per 1000 ECMO days, our results are in the high range of previously published studies, probably linked to the severity of our patients (SAPS 2 score at admission of 54.4 (22.3)) and to the extensive review of hospitalization reports, including daily observation, biological and microbiological data.

Moreover, since cardiopulmonary bypass (CBP) is know to induce an immunosuppression, decreasing monocyte response to pathogens [7–9] and significant reduction of HLA-DR and TLR4 expression on alveolar macrophages [16], ECMO could induce the same alterations that CBP and thus predispose patients to infectious complications, in particular pneumonia. However, to the best of our knowledge, there is no pre-clinical data to support this hypothesis so far. Finally, it is interesting to note that the main microorganisms documented in our cohort were Enterobacteriaceae (39.4%) and *Pseudomonas aeruginosa* (18.2%), even though pneumonia was an early event during assistance. This proportion is similar to that found in the post cardiac surgery context [17], and could be linked with a bacterial translocation in this context of circulatory failure.

In our cohort, Renal Replacement Therapy (RRT) and documentation of *Pseudomonas aeruginosa* were associated with treatment failure. The increase of volume of distribution as much as the antibiotic drug sequestration in the circuit secondary to ECMO could accentuate the pharmacokinetic alterations seen in critically ill patients, leading to poor achievement of target concentrations of antibiotics, especially hydrophilic antibiotics used against *P*. *aeruginosa* (e.g., aminoglycosides, beta-lactams) [18]. Moreover, RRT is known to add a significant variability in antibiotic concentrations [19,20]. Hence, it was shown that the combination of ECMO and RRT could alter meropenem pharmacokinetic, especially for *P*. *aeruginosa* [11].

Our study suffers from several limitations. The diagnosis of VAP is challenging for clinicians [21], and even more in patients assisted with ECMO, since the parameters usually used for VAP diagnosis are not interpretable in this setting [22,23]. Hence, to our knowledge, the diagnosis criteria of pneumonia are not specified in any of the above studies about infections under ECMO support. In our study, VAP was defined *post hoc* by 2 independent investigators (AB, CB) on the basis of clinical and radiological data, and systematically confirmed with a positive quantitative culture of a respiratory sample at significant threshold. Although all patients assisted with VA-ECMO between January 2013 and December 2014 were included and analyzed, the retrospective design of the study as well as the relatively low sample size of the cohort limits the strength of our findings. In addition, even though SOFA score was significant in the univariate analysis of risk factors for pneumonia, the large number of missing data did not allow us to integrate it into the multivariate analysis. Finally, the monocentric design of the study limits its external reproducibility, even if a large cohort obtained on a short period and in a single center guarantees homogeneity of management.

## Conclusion

VAP is a frequent and severe complication in patients treated with VA-ECMO, and is associated with an increased morbidity and mortality. RRT during antibiotic therapy and infection by *Pseudomonas aeruginosa* appear as strong risks factors of treatment failure. The best antibiotic management in patients with RRT and with ECMO remains to be determined.

## Supporting information

S1 Table Characteristics at VAP diagnosis. (DOCX)

S2 Table Diagnosis criteria at VAP diagnosis. (DOCX)

S1 Fig Definitions of persistence, relapse and superinfection. (DOCX)

S2 Fig Evolution of clinical and biological parameters between D0 and D14 depending on the presence (grey line) or absence (blue line) of a VAP recurrence. a) Leukocytosis, b) PCT serum level c) FiO2 ventilator, d) PaO2, e) FiO2 ECMO, f) ECMO circuit blood flow, g) ECMO sweep gas flow, h) Temperature. Data are mean value (± standard deviation).
(DOCX)

S1 File Management of patients under ECMO support. (DOCX)
