# Supplementary material for: Ventilator-associated pneumonia in patients assisted by veno-arterial extracorporeal membrane oxygenation support: Epidemiology and risk factors of treatment failure
Source: PLoS One. 2018 Apr 13;13(4):e0194976. doi: 10.1371/journal.pone.0194976 (PMC5898723; doi:10.1371/journal.pone.0194976)
Supplement: S1 Table — (DOCX) [file pone.0194976.s001.docx]

**S1 Table: Characteristics at VAP diagnosis**

| **Characteristics** | | **Results** |
| --- | --- | --- |
| Prophylactic antibiotic treatment at implantation | | 67.1% |
| Previous antibiotherapy during ICU stay | | 34.9% |
| Blood lactate (mmol.L^-1^) | | 2.7 (2.6) |
| Proton Pump Inhibitor | | 83.7% |
| Neuromuscular blockade | | 5.8% |
| Amines | Epinephrine | 44.7% |
|  | Norepinephrine | 10.6% |
|  | Dobutamine | 10.6% |
|  | Noradrenaline + dobutamine | 21.2% |
| Oxygenation | PaO_2_ (mmHg) | 122.4 (59.8) |
|  | FiO_2_ ventilator (%) | 59.9 (23.9) |
|  | FiO_2_ ECMO (%) | 74.2 (29.0) |
|  | ECMO flow rate (L.min^-1^) | 3.8 (1.4) |
|  | Sweep gas flow rate (L.min^-1^) | 3.6 (2.1) |
| Nutrition | Parenteral | 14.8% |
|  | Enteral | 54.3% |

Data are No. (%) of patients or mean value (± standard deviation).
