# Supplementary material for: Ventilator-associated pneumonia in patients assisted by veno-arterial extracorporeal membrane oxygenation support: Epidemiology and risk factors of treatment failure
Source: PLoS One. 2018 Apr 13;13(4):e0194976. doi: 10.1371/journal.pone.0194976 (PMC5898723; doi:10.1371/journal.pone.0194976)
Supplement: S2 Table — (DOCX) [file pone.0194976.s002.docx]

**S2 Table: Diagnosis criteria at VAP diagnosis**

| **Diagnosis criteria** | **No (%)** |
| --- | --- |
| **Clinical criteria** | |
| Temperature > 38°C | 12 (14.0) |
| Temperature < 36°C | 42 (48.9) |
| Purulent secretions | 39 (45.3) |
| **Biological criteria** | |
| Leukocytosis > 12,000/mm^3^ | 62 (72.1) |
| Leucopenia < 4,000/mm3 | 8 (9.3) |
| Serum procalcitonin > 0.5 ng/mL | 77 (94.6) |
| **Radiological criteria** | |
| Radiographic infiltrate | 66 (76.7) |
| *Right* | 34 (39.5) |
| *Left* | 5 (5.8) |
| *Bilateral* | 27 (31.4) |
| Pleural effusion | 24 (27.9) |

Data are No. (%) of patients or mean value (± standard deviation).
