# Supplementary figures and images for: Ventilator-associated pneumonia in patients assisted by veno-arterial extracorporeal membrane oxygenation support: Epidemiology and risk factors of treatment failure
Source: PLoS One. 2018 Apr 13;13(4):e0194976. doi: 10.1371/journal.pone.0194976 (PMC5898723; doi:10.1371/journal.pone.0194976)

**
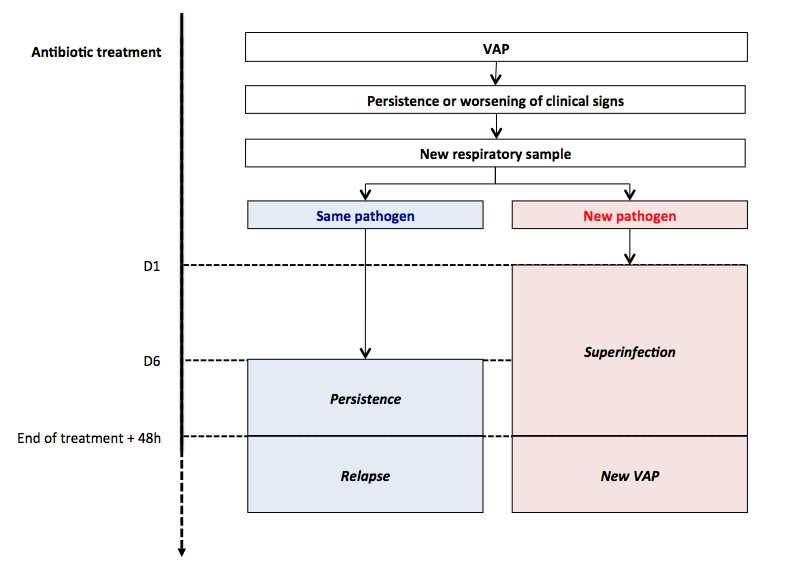
**

Supplement: S1 Fig — (DOCX) [file pone.0194976.s003.docx]

**
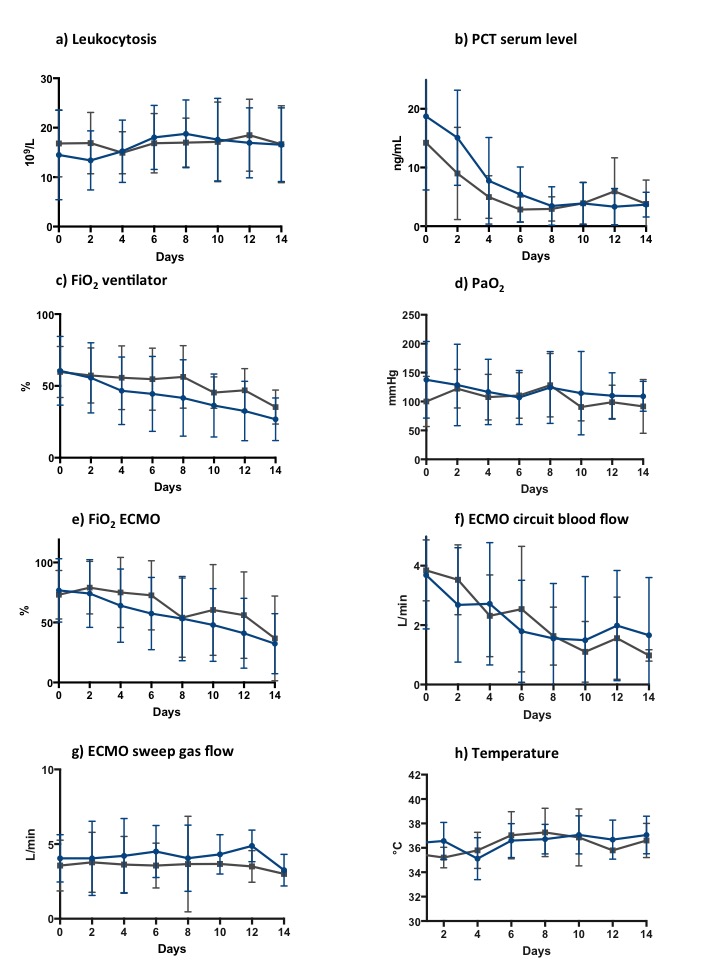
**

Supplement: S2 Fig — a) Leukocytosis, b) PCT serum level c) FiO2 ventilator, d) PaO2, e) FiO2 ECMO, f) ECMO circuit blood flow, g) ECMO sweep gas flow, h) Temperature. Data are mean value (± standard deviation). (DOCX) [file pone.0194976.s004.docx]
