# Supplementary material for: Ventilator-associated pneumonia in patients assisted by veno-arterial extracorporeal membrane oxygenation support: Epidemiology and risk factors of treatment failure
Source: PLoS One. 2018 Apr 13;13(4):e0194976. doi: 10.1371/journal.pone.0194976 (PMC5898723; doi:10.1371/journal.pone.0194976)
Supplement: S1 File — (DOCX) [file pone.0194976.s005.docx]

**S1 File: Management of patients under ECMO support**

*ECMO management*

Procedures for implantation, daily management and weaning were realized according to according to the recommendations of the Extracorporeal Life Support Organization (ELSO) as follows. VA-ECMO was implanted in patients with a refractory cardiogenic shock resulting on right, left or biventricular failure. According to the etiology and the degree of emergency, the procedure was realized in the pre-, per- or post-cardiotomy period, at the patient's bed or in the operating room. Prophylactic antibiotic therapy was performed with second-generation cephalosporin as a single intravenous injection (cefazoline 2g).

Peripheral veno-arterial ECMO cannulation was mainly used: blood is drained via a cannula from the right atrium, oxygenated and decarboxylated in a dedicated extracorporeal rotor/oxygenator device and returned via a second cannula to the descending aorta through peripheral cannulation of femoral artery. Alternative peripheral sites may also be used such as axillary or jugular vessels. Cannulae were usually placed percutaneously by the Seldinger technique, or surgically in open technique.

For central ECMO, the right atrium and the ascending aorta are used as vascular access, with the former for drainage and the latter for supply. This type of ECMO is rather used in the post-cardiotomy period, or in patients with extensive atherosclerosis. The peripheral cannulation was switched to central if acute leg ischemia or acute pulmonary edema due to left ventricular insufficiency occurred.

An anticoagulant treatment with unfractionated heparin was established in order to obtain an anti-Xa activity between 0.2 and 0.4 IU/mL, except in the case of haemorrhagic complication, where treatment was interrupted.

The ECMO weaning potential was assessed daily according to the recommendations of the Extracorporeal Life Support Organization (ELSO). Weaning was considered after optimization of inotropic drugs: 1) in the objective presence of electrical pulsatility and echocardiographic contractility; 2) in the absence of hemodynamic deterioration after a 50% and then 25% assistance rate reduction of the initial flow, and; 3) in the absence of hemodynamic deterioration after a clamping test lasting from 30 minutes to 4 hours.
